# Supplementary material for: The ATF3–OPG Axis Contributes to Bone Formation by Regulating the Differentiation of Osteoclasts, Osteoblasts, and Adipocytes
Source: Int J Mol Sci. 2022 Mar 23;23(7):3500. doi: 10.3390/ijms23073500 (PMC8998270; doi:10.3390/ijms23073500)
Supplement: Supplementary file 1 [file ijms-23-03500-s001.zip › ijms-1624013-supplementary-done.pdf]

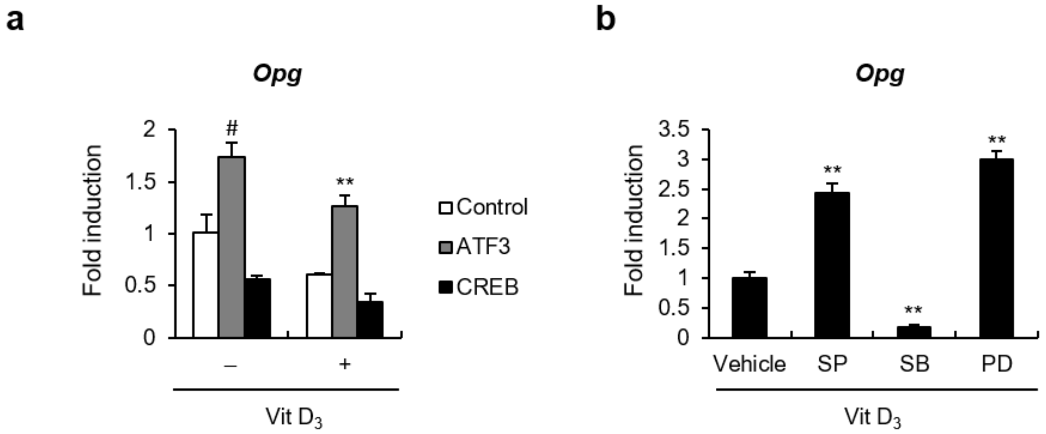


**Figure S1. The effect of p38$\alpha$-CREB axis on OPG transcription in primary osteoblast precursors.** (a) Primary osteoblast precursors transduced with pMX-IRES-EGFP (control), ATF3, or CREB retrovirus were cultured in the presence or absence of Vit $D_3$. (b) Primary osteoblast precursors were treated with DMSO (Vehicle), SP600125 (5 μM), SB203580 (10 μM), or PD98059 (20 μM) and further cultured in the presence of Vit $D_3$. *Opg* expression was analyzed by real-time PCR. # $p < 0.05$, ** $p < 0.001$ vs. control.

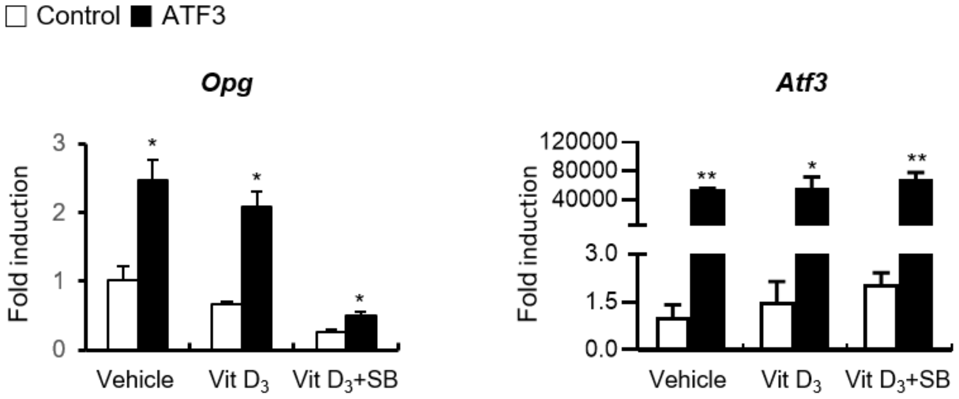


**Figure S2. ATF3 overexpression slightly recovers the reduced *Opg* expression by SB203580.** Primary osteoblast precursors transduced with pMX-IRES-EGFP (control) or ATF3 retrovirus were cultured in the presence or absence of SB203580 and Vit $D_3$. Gene expressions of the indicated genes were analyzed by real-time PCR. * $p < 0.01$, ** $p < 0.001$ vs. control.
